# Colorado’s first year of extreme risk protection orders

**DOI:** 10.1186/s40621-021-00353-7

**Published:** 2021-10-20

**Authors:** Leslie M. Barnard, Megan McCarthy, Christopher E. Knoepke, Sabrina Kaplan, James Engeln, Marian E. Betz

**Affiliations:** 1 Department of Epidemiology, Colorado School of Public Health, Leslie Barnard, 3438 N Gilpin Street, Denver, CO 80205 USA; 2 Department of Emergency Medicine, School of Medicine, University of Colorado Anschutz Medical Campus, Aurora, CO USA; 3 Adult & Child Consortium for Outcomes Research & Delivery Science, School of Medicine, University of Colorado Anschutz Medical Campus, Aurora, CO USA; 4 Division of Cardiology, School of Medicine, University of Colorado Anschutz Medical Campus, Aurora, CO USA; 5 Denver Health Emergency Medicine Residency, Denver Health Medical Center, Denver, CO USA; 6 VA Eastern Colorado Geriatric Research Education and Clinical Center, Aurora, CO USA

**Keywords:** Epidemiology, Injury, Firearm

## Abstract

**Background:**

Extreme Risk Protection Orders (ERPOs) are a relatively new type of law that are being considered or implemented in many states in the United States. Colorado’s law went into effect on January 1, 2020, after significant controversy and concern over potential misuse of the law to confiscate weapons; many (*n* = 37 of 64) counties declared themselves “2nd Amendment (2A) sanctuaries” and said they would not enforce the law. Here, reviewed the patterns of use of the law during its first year.

**Methods:**

We obtained all court records for ERPO petitions filed between January 1 and December 31, 2020. Data elements were abstracted by trained staff using a standardized guide. We calculated the proportion of petitions that were approved or denied/dismissed, identified cases of obvious misuse, and examined patterns by 2A county status.

**Finding and results:**

In 2020, 109 ERPO petitions were filed in Colorado; of these, 61 were granted for a temporary ERPO and 49 for a full (year-long) ERPO. Most petitions filed by law enforcement officers were granted (85%), compared to only 15% of petitions filed by family or household members. Of the 37 2A sanctuary counties, 24% had at least one petition filed, versus 48% of non-2A sanctuary counties. Across the 2A counties, there were 1.52 ERPOs filed per 100,000 population, compared to 2.05 ERPOs filed per 100,000 in non-2A counties. There were 4 cases of obvious law misuse; none of those petitions resulted in an ERPO or firearm confiscation.

**Conclusion:**

State-level studies suggest ERPOs may prevent firearm injuries. Robust implementation, however, is critical for maximal effect. Understanding ERPO experiences and challenges can inform policy creation and enaction in other states, including identifying how best to address concerns and facilitate evaluation.

## Background

In 2019, there were 39,707 firearm-related deaths in the United States, 60% from suicide and 36% from homicide; (CDC 2021) in the same year in Colorado, there were 846 firearm related deaths, 75% of them due to suicide (Stats of the States 2021). Reducing firearm access during times of risk of suicide or interpersonal violence is recommended, including through voluntary, temporary out-of-home firearm storage, secure storage at home (SurgeonGeneral. 2012; Allchin et al. 2019), or through legal tools which limit individuals’ access to firearms.

Extreme Risk Protection Orders (ERPO) are one such tool (Extreme Risk Laws 2021). These civil orders provide a process for temporary removal of firearms from individuals who threaten imminent violence against themselves or others. State-level studies suggest ERPOs may prevent firearm suicides (Kivisto and Phalen 2018; Swanson et al. 2019; Swanson, et al. 2017), and there are documented cases where ERPOs have been used to prevent mass shootings (Wintemute et al. 2019). Importantly, ERPOs are not intended to target or stigmatize individuals living with mental illness, but rather those who pose a specific risk of violence. Mental illness rarely contributes to violence, especially interpersonal violence. (Elbogen et al. 2016; Swanson et al. 2006; Dorn et al. 2012) As of 2021, ERPOs have been passed and enacted in 19 states and the District of Columbia; the COVID-19 pandemic spike in firearm purchases (Schleimer et al. 2021) and interpersonal violence (Abdallah et al. 2021) increased attention to ERPOs and discussion of federal legislation (McBath 2019).

In Colorado, as in many states, a court reviews the petition to first deny or grant a temporary ERPO (TERPO, up to two weeks); if the TERPO is granted, the court holds a hearing to determine whether to deny or grant a 364-day ERPO, also called a final ERPO. Petitioners must demonstrate with clear and convincing evidence (threats made publicly, privately, or through social media or other forms of communication) that a respondent poses a significant risk to self or others by having a firearm. If a TERPO/ERPO is granted, the respondent must surrender firearms and concealed carry licenses and may not acquire firearms during the specified period. Once a TERPO/ERPO is vacated or expires, the respondent may reclaim firearms and acquire new ones, subject to other possession requirements.

Colorado’s ERPO law is similar to laws in other states related to who can petition for firearm removal (family members or law enforcement) and why (Colorado General Assembly 2021). The use of TERPOs followed by a final ERPO hearing is also not unique to Colorado, nor is the ability of petitioners to file against respondents who do not currently possess firearms as a way to prevent them from purchasing or acquiring firearms. However, Colorado’s ERPO statute is unique in requiring law enforcement petitioners to concurrently file a search warrant for firearms in possession of the respondent. Also, in Colorado family members (rather than law enforcement) may remove and keep the firearms from the respondent.

Colorado’s ERPO is also notable in the controversy surrounding its 2019 passage, in part due to fears of misuse as a way to unfairly confiscate firearms (Sanchez 2021). Over half of Colorado counties (37 of 64) declared some form of “Second Amendment (2A) sanctuary” status (Colorado and counties have declared themselves 2021), such as sheriffs or county commissioners stating they would not enforce the law (e.g., by not filing petitions or by not removing firearms for granted petitions). When Colorado’s law went into effect, information was posted on state webpages, such as the Colorado Judicial Branch website (Branch et al. 2021) and the Colorado General Assembly website (Colorado General Assembly 2021) but there was no large-scale public education campaign. The constitutionality of the ERPO law was challenged – but upheld (Boyer 2012)—in Colorado state court before the law went into effect on grounds of violating the Second Amendment, due process, and search and seizure.

The experience of ERPO passage in Colorado led to questions about how the law was being used, or misused, across counties. Recent analyses of data from California (Pallin et al. 2020),Washington (Morgan et al. 2018), and Oregon (Zeoli et al. 2021) found the majority of petitions were filed by law enforcement. In California, there has been significant county-level variation in law usage and in Washington, most petitions were among threats to others alone, followed by both self and others and then self alone. Here, we sought to examine the first year of TERPOs/ERPOs in Colorado to describe patterns of petitions, granted TERPOs/ERPOs, and potential misuse, including in 2A counties.

## Methods

### Sample

We collected and examined all court records from the first year of Colorado’s ERPOs (January 1—December 31, 2020) (Colorado General Assembly 2021). Staff were trained to abstract documents to a secure, centralized database based on a coding guide (inter-rater reliability: 85% of a random 10% sample). Any disagreements in coding were adjudicated by a third research staff member.

Abstracted data elements included: respondent demographics (age, gender, race, ethnicity, county of residence); identity of petitioner (family or household member, versus law enforcement officer (LEO)); reason for petition being filed (threats against self, others or both), reasons for petitions being granted or denied (as reported via checkbox in court documents), if the respondent had a documented mental health issue, if there was documentation of firearms removed by or turned in to LEOs and/or returned to the respondent, misuse was defined as the petitioner falsely characterized their relationship to the respondent and malicious intent of petitioners defined as being charged with perjury. Forms varied somewhat by county, including in what data (if any) were redacted, so all elements were not available for all cases.

### Analysis

We first used descriptive statistics (number and proportion) to summarize the demographic characteristics of respondents and the numbers and reasons for petitions being granted or denied We calculated rates using 2020 US census data for population denominator. We then compared the patterns of petition filing and granting by various subgroups, including race and ethnicity, petitioner identity, and 2A county status. Since county-level data regarding firearm ownership are not available in Colorado, we followed prior work (Azrael et al. 2004; Rowhani-Rahbar et al. 2017) and created a proxy estimate by calculating the ratio of firearm suicides to total suicides for 2A and non-2A counties (with this ratio generally being higher when firearms are more prevalent). Finally, we examined potential misuse of the law and defined misuse as the petitioner filing a fictitious petition (e.g., claiming a false relationship or reason for petition). This study was deemed exempt by the Colorado Multiple Institutional Review Board.

## Finding and results

We obtained records for all petitions filed in 2020 (*n* = 109). We excluded cases missing key case facts or outcomes (*n* = 11; 10%), such as where the final order hearing was missing so the petition outcome (and rationale) could not be identified. We also excluded duplicates where the petitioner, respondent, and facts matched (*n* = 12; 11%), such as when a petitioner filed the same petition more than once.

### Patterns of use

**Fig. 1:**
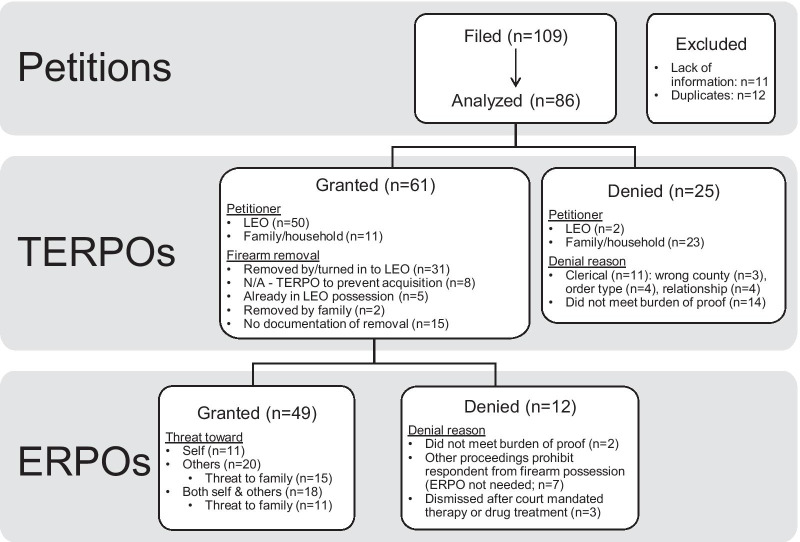
Characteristics and outcomes of ERPO petitions, TERPOs, and ERPOs (Colorado, 2020)

Of the 86 petitions remaining for full analysis, 25 (29%) were denied and 61 (71%) received two-week TERPOs; of those, 49 (80%) continued into 364-day ERPOs and the remaining 12 petitions that received TERPOs were ultimately denied (Fig. 1).

The majority of petitions were filed for risk of harm against others (50 filed, 20 granted full 365-day ERPOs), followed by both self and others (25 filed, 18 granted) and against self alone (11 filed, 11 granted). Redacted or missing birth dates precluded calculation of age, but no respondents were identified as juveniles. Most petitions were against respondents who were males (*n* = 73; 85%) and non-Hispanic whites (*n* = 59; 80%). Among all male respondents, 42 (58%) had petitions granted (versus 6 [75%] of female respondents). Among non-Hispanic white respondents, 39 (66%) had petitions granted (versus 7 [64%] of respondents identified as black or other/unknown). Among those where a TERPO was granted, white respondents were overrepresented (84% of TERPOs versus 69% in state population (Race and (Census Tracts) 2021)) while racial and ethnic minorities (REM) were underrepresented (16% versus 31%). Among counties with 2A status, REM were underrepresented (23% of petitions filed vs 26% of the population) and among non-2A counties this underrepresentation was even more pronounced (17% of petitions filed vs 34% of the population).

Most TERPOs (*n* = 50; 82%) and subsequent year-long ERPOs (*n* = 44; 85%) filed by LEOs were granted. Fewer TERPOs (*n* = 11, 18%) and year-long ERPOs (*n* = 5, 15%) filed by family or household members were granted. Of non-LEO petitioners who had TERPOs granted, five were partners sharing a child with the respondent; others were in a domestic partnership (*n* = 2), a parent–child relationship (*n* = 2), or lived with (*n* = 2) the respondent.

Of the 61 cases where a TERPO was granted, 31 (51%) had documentation that firearms were removed by or turned in to LEOs (Fig. 1). Of the remaining granted TERPOs, 8 (16%) were against individuals who did not currently possess firearms as a way to keep respondent from purchasing firearms, 5 (8%) had other protections (e.g., criminal charges) already in place that removed the firearms, and 2 (3%) had other people (e.g., family members) who removed the firearms without police involvement. There were fifteen cases without documentation of firearm removal; in one case, it was noted that documentation was difficult owing to the respondent experiencing homelessness, but the remaining 14 (25%) of cases did not provide additional information. Among the four TERPOs with firearm removal and subsequent ERPO denial, three (75%) had documentation of firearms being returned to the respondent; in the fourth case, other ongoing criminal proceedings prohibited firearm return.

Across all counties, three petitions were denied because the petitioner filed in their own county of residence instead of the respondent’s. In four cases, petitions were denied because there was no firearm-specific information provided, and a general restraining order or non-firearm protection order was more appropriate. Seven petitions were dismissed due to another law or legal proceeding that already prohibited the respondent from possessing firearms.

### Cases of misuse

There were four instances of misuse where the petitioner falsely characterized their relationship to the respondent. All four were denied. One case was considered to be of malicious intent and led to perjury charges against the petitioner. Petitioners in the other three cases were not charged: one was already incarcerated and had filed against prison guards; one appeared to have misunderstood law requirements and filed against a neighbor; and one filed against an entire police department with evidence of mental illness of the petitioner who claimed to live with the police department. We found no cases of malintent when the petitioner was legally allowed to petition; there were cases where the petitioner did not meet the burden of proof, but no perjury charges were filed against the petitioner in any of these cases.

### Use in 2A counties

**Fig. 2:**
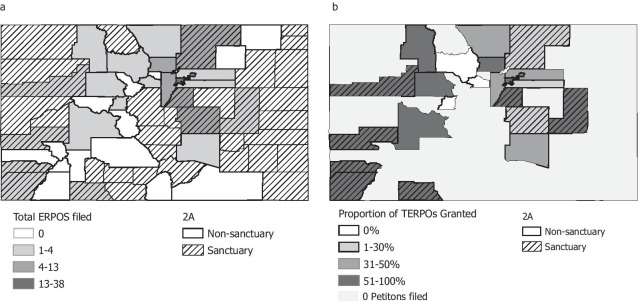
ERPO petitions filed in 2020 in Colorado, by county and Second Amendment Sanctuary status: **a** total petitions and **b** proportion of petitions with granted TERPO

Of the 37 counties who self-declared a 2A sanctuary status, 9 (24%) had at least one petition filed, versus 13 (48%) of counties without sanctuary status (Fig. 2). Across the 2A counties, versus non-2A counties, there were lower rates of ERPO petitions filed and of petitions being granted, although the small sample size precluded testing for statistical significance. In 2A counties, there were 1.52 ERPOs petitions filed per 100,000 population; among these petitions, 48% were granted for TERPOs and 36% for full ERPOs. In non-2A counties, there were 2.05 ERPO petitions filed per 100,000 (80% granted for TERPOs and 66% for ERPOs). 2A sanctuary counties had a lower proportion of petitions filed by LEOs (35% versus 53% in non-2A counties), but these were still more likely to be granted (73%) versus those filed by non-LEOs (5%). Of the granted TERPOs in 2A sanctuary counties (*n* = 12), ten (83%) had evidence of firearms being removed, and nine were continued into 365-day ERPOs (75%). Based on public data, a greater proportion of suicides are completed by firearm in 2A (56%) versus non-2A (48%) counties in Colorado, suggesting a higher population prevalence of firearms. The number and rate of concealed carry permit applications in 2020 was also higher in 2A counties (2A: 26,067, 0.014 per 100,00 population; non-2A: 21,972, 0.006 per 100.00 population).

## Discussion

In 2020 in Colorado, 61 TERPOs and 49 ERPOs were granted in a state with 5.6 million residents (rate: 1.09 and 0.88 per 100,000 population). This is lower than the rate of usage in California in 2019 (717 granted; rate 1.82 per 100,000 population (Pallin et al. 2020)) or Maryland (8.2 per 100,000) (Baltimore Sun. 2021). Colorado’s law implementation occurred during a year marked by the COVID-19 pandemic and state stay-at-home orders, in addition to other restrictions. In all counties, records had to be filed in person, potentially contributing to a lower rate of use than expected or needed.

Despite public concern about inappropriate or malicious use and firearm confiscation, only four of 86 petitions (4.7%) in Colorado in 2020 were obviously misused in terms of the relationship listed on the petition, one was determined to be filed with malintent, but of these ERPOs were granted. Notably, no petitions filed by someone legally allowed to petition under Colorado law (i.e., spouses/partners, or former partners with whom respondents share a child, other family members, or law enforcement) were denied owing to apparent malice by being charged with prudery. There were, however, petitions dismissed because of filing errors, such as filing in the wrong county. Other cases were dismissed because of inadequate evidence; from this analysis alone, we cannot tell whether these dismissals were appropriate (i.e., there was not evidence to support the request) or reflected an incomplete petition (i.e., evidence existed but was not provided in the petition). The level of evidence provided may have contributed to why most TERPOs/ERPOs filed by LEOs were granted compared to non-LEO filings, if LEO petitions had more robust evidence, such as reference to prior law enforcement reports. Similar to Oregon (Zeoli et al. 2021), our findings suggest the need for further education of the public and LEO to guide the public about how, when, and where to file petitions.

Across the U.S., state and county officials have passed 2A sanctuary declarations. Colorado’s ERPO passage was marked by significant controversy, with numerous counties declaring 2A sanctuary status. These are largely symbolic, non-binding and hold no legal standing (Turret et al. 2020). The constitutionality of such declarations and legislation is unclear. In the first year of ERPO cases in Colorado, there were petitions both filed and granted in 2A counties, and both types of counites had firearms removed and returned. However, the rates of filing and granting appeared lower in 2A than in non-2A counties; the differences are even greater when accounting for the higher rate of firearm ownership or usage in 2A counties. It is possible that some ERPO cases were so compelling that local officials broke their commitment to the 2A sanctuary status, or that entire counties were not united or bound by a sheriff’s or official's declaration, so petitions could be filed and approved without their input. Future evaluations, as cases accumulate, may identify statistically significant patterns between 2A and non-2A counties, as well as change over time. One potential ramification of 2A counties or states is a chilling effect on petitioning, especially for ex-parte petitioners who may incorrectly think that ERPOs aren’t legal in their jurisdiction and elect not to pursue them in appropriate cases. in their jurisdiction and elect not to pursue them in appropriate cases. Declaring one’s locality a “Second Amendment Sanctuary” does not reduce firearm suicides or other forms of violence. These declarations are not legally-binding, and our analysis highlighted instances in which such counties enforced ERPOs, and this finding may support a public information campaign or other outreach efforts may encourage people in these counties to petition for orders when someone is at risk of violence or efforts to repeal such 2A sanctuary declarations and/or legislation in the interest of public safety (Ulrich 2019).

A limitation of our study is that records were often incomplete in details on firearms being removed by or turned in to LEOs, and there were no available details about *how* firearms were removed or whether threats or harm occurred to LEOs during the process. The relatively small number of petitions included in this analysis may lead to unstable rates and percentages, so future studies using multiple years and pooled data will be important to identify statistical differences or trends, including comparison of Colorado’s usage to other states. Additional challenges included variability in county processes for requesting and obtaining documents, which may complicate future work or ongoing tracking.

ERPOs may play a role in suicide and firearm injury prevention in Colorado. The Colorado state legislature passed a law in 2021 establishing an Office of Gun Violence Prevention (Hansen et al. 2021) tasked with conducting public awareness campaigns. These results could be leveraged by this office, policymakers and those implementing ERPOs in CO by including more robust public education about ERPO resources available to them. We found little evidence of misuse as an unintended consequence. Despite controversy surrounding the law, petitions were granted for 49 cases where threats against self or others was demonstrated. Understanding ERPO experiences and challenges can inform policy creation and enaction in other states, including identifying how best to address concerns and facilitate evaluation.

## Data Availability

All data are publicly available upon request: https://www.courts.state.co.us/Forms/Forms_List.cfm?Form_Type_ID=280.
